# Improving the Acceptance Rate of Centchroman As a Postpartum Contraceptive Through a Quality Improvement Initiative

**DOI:** 10.7759/cureus.29277

**Published:** 2022-09-17

**Authors:** Avir Sarkar, Isha Wadhawan, Anjaly Raj, Prerana Nagabhushana, Preeti Singh

**Affiliations:** 1 Obstetrics and Gynaecology, Employees' State Insurance Corporation (ESIC) Medical College and Hospital, Faridabad, IND; 2 Obstetrics and Gynaecology, All India Institute of Medical Sciences (AIIMS), New Delhi, IND; 3 Obstetrics and Gynaecology, Jawaharlal Institute of Postgraduate Medical Education and Research, Puducherry, IND

**Keywords:** oral contraception, oral contraceptive pill (ocp), temporary contraception, birth spacing, quality improvement, postpartum contraception, centchroman

## Abstract

Background

The unmet need for contraception is two-pronged: a spacing method and a permanent method. Centchroman, a non-hormonal, non-steroidal oral contraceptive, is suitable for both of these purposes. The Government of India provides it free of cost under the name "Chhaya", but its current acceptance rates are lower than expected. We aimed to increase the acceptance rates of centchroman as a postpartum contraceptive through a quality improvement (QI) approach conducted over eight months at a tertiary care hospital in North India.

Materials and Methods

This QI study was done in three phases: a pre-intervention phase of over eight weeks to assess the baseline acceptance and prevalence rates of centchroman use; an intervention phase of over 12 weeks involving three Plan-Do-Study-Act (PDSA) cycles to increase the awareness and acceptance of centchroman among the target population using visual aids and counselling by the nursing staff and resident doctors, respectively; and a post-intervention phase of over 12 weeks to assess the acceptance and continuation rates of Chhaya.

Results

The acceptance rates for centchroman increased from a baseline of 2.9% to 15.3%, 56.3%, and 78.2% after the first, second, and third PDSA cycles, respectively. On follow-up, continuation rates were 96.7%, 89.5%, and 78.6% at one, three, and six months, respectively. The majority of women reported only minor side effects, with the primary reason for discontinuation being a preference for intrauterine devices or medroxyprogesterone acetate injections over Chhaya.

Conclusion

The postpartum period provides an important window of opportunity to counsel women for contraception. Despite an enviable safety profile and dosing schedule, centchroman remains largely under-utilized. Increasing awareness among women as well as health care workers may improve the acceptance of centchroman and help reduce the burden of untimely and unwanted conceptions.

## Introduction

Birth spacing is the cornerstone for good outcomes in obstetrics. Effective and regular use of contraception reduces unintended pregnancies, thereby minimising complications associated with medical termination of pregnancy and pregnancy-related morbidity and mortality, which, in turn, improves the social and economic status of women in society. Even though the recent data from the Fifth National Family Health Survey report suggests an improvement in the total unmet need for contraception to 9.4% (four per cent is the unmet need for spacing), it is as high as 28.2% in some areas of the country. The ideal contraception method should be inexpensive, easily accessible, safe with few side effects, non-interfering with sexual drive, highly effective, reversible, and reusable. Despite being a non-hormonal and non-steroidal form of contraception, centchroman has not gained popularity as a postpartum contraceptive [1]. It was approved in 1991 and was first launched into the Family Planning Program by the Government of India in 1992. It acts by hastening the transport of zygotes from the fallopian tube to the uterus and suppressing endometrial proliferation, thus affecting the process of implantation [2]. There is no interference with ovulation or any effects on the hypothalamic-pituitary-ovarian axis [3]. It is safe during breastfeeding and has not shown any major side effects during lactation [1]. The Government of India is providing it free of cost under the name "Chhaya". Over the years, it was found that despite a good overall acceptance rate of postpartum contraception (around 67% according to the Fifth National Family Health Survey report), Chhaya remained a neglected option in our institute (2.9% acceptance rate).

There are very few studies available regarding the acceptance rates of centchroman in India. The acceptance rates widely range from 60 to 77.5% [1,4]. However, no literature exists on the contraceptive acceptance rate of centchroman in the postpartum period. No previous studies were conducted to increase its acceptance among mothers in the postpartum period. Hence, a quality improvement (QI) approach was initiated to improve the acceptance rates of centchroman among women visiting postnatal clinics six to eight weeks after childbirth.

## Materials and methods

This QI initiative was conducted at the Employees' State Insurance Corporation (ESIC) Medical College and Hospital, Faridabad, a tertiary care hospital in North India for a period of eight months. It aimed to increase the acceptance rates of centchroman as a postpartum contraceptive among women visiting the clinic for a postpartum checkup. The approval of the Institutional Ethics Committee of Employees' State Insurance Corporation (ESIC) Medical College and Hospital, Faridabad, was obtained prior to the commencement of the study (IRB-197/2021). The study was conducted in three phases: a pre-intervention baseline phase (eight weeks), an intervention phase (12 weeks), and a post-intervention follow-up phase (12 weeks).

**Figure 1:**
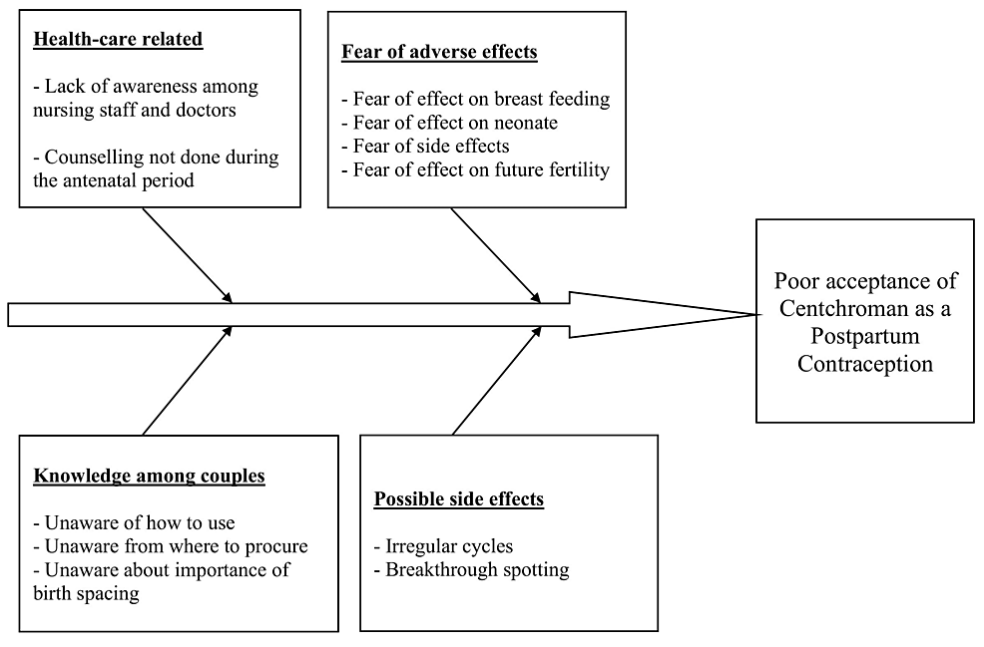
A fishbone analysis of the possible reasons for low acceptance rates of centchroman as a postpartum contraceptive The figure has been created by the authors.

During the pre-intervention baseline phase, a baseline audit was conducted to assess the prevalence of centchroman use among women in the postpartum phase visiting postnatal clinics. A QI team was constituted with six members under the guidance of a team leader. Team members included faculty, resident doctors, and nursing staff working in postnatal clinics. The dedicated QI team formulated the problem statement, smart aim, and strategies to implement interventions. The acceptance rate of centchroman as a postpartum contraceptive was quite low. To elicit the problem, the following questions were asked: whether the patient understood the need for contraception, whether she had previously been approached by health care workers and offered contraception counselling, what options she was given, and whether she was aware of centchroman as an effective postpartum contraception option. The problem statement tried to address the low acceptance rate of centchroman among the participants of the study. The smart aim was to increase the acceptance rates to at least 50% through a three-phase QI initiative by creating awareness using visual aids and counselling by resident doctors and nursing officers. Measures included continued awareness programmes and counselling by health care workers regarding centchroman. Baseline data was collected over eight weeks to identify the existing acceptance rates and prevalence of the use of centchroman as a postpartum contraceptive. The baseline data was collected from the contraceptive audits that are done frequently in family planning outpatient departments every week. After the collection of baseline observations, a second team meeting was conducted, and the possible reasons for the low acceptance of centchroman were discussed. A conceptual framework of the possible reasons for the low acceptance rates of Centchroman as postpartum contraception was discussed and the same was plotted in the form of a fishbone analysis chart (Figure 1). A total of three Plan-Do-Study-Act (PDSA) cycles were proposed to improve the acceptance of centchroman in the postpartum period.

The intervention phase consisted of three PDSA cycles. Before starting the first intervention, a seminar was conducted that focused on the training and education of the health care workers to increase awareness regarding the advantages of centchroman as a method of non-hormonal postpartum contraception. In the first PDSA cycle, multiple charts depicting the advantages, side effects, and dosing schedules of centchroman were displayed in the postnatal clinics. In the second cycle, awareness regarding centchroman was created by the nursing staff on duty among women who were in their postpartum phase. The women were educated on the role of centchroman as a spacing method. During the third cycle, resident doctors posted in the postnatal clinics were involved in the counselling of women. New mothers were counselled about the benefits of this non-hormonal method of contraception. Each cycle lasted for four weeks. The primary outcome was the increase in acceptance of centchroman as a postpartum contraceptive. Data collectors (six team members) maintained records of the number of women counselled and those who opted in for centchroman. Follow-up observations in the post-intervention phase were collected for 12 weeks to assess the continuation rate of Chhaya. During this period, only observation was done without any intervention. All data were entered in a Microsoft Excel matrix and analysed using IBM Statistical Package for Social Sciences (SPSS) Statistics version 20.0.0. Categorical variables were expressed in percentages and frequencies.

## Results

**Figure 2:**
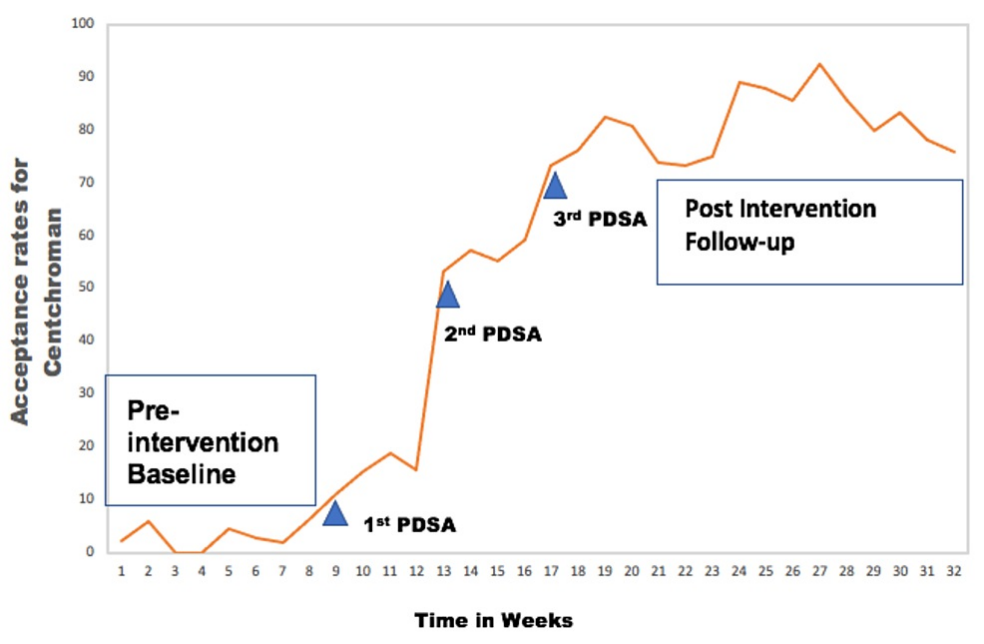
Figure showing the increase in the rate of acceptance of centchroman as a postpartum contraceptive with the implementation of each PDSA cycle across time The figure has been created by the authors.

Baseline observations demonstrated poor acceptance of centchroman among new mothers who were offered various options for contraception at the postnatal clinic. The reasons for this could be that centchroman was not initially offered by most clinicians due to a lack of awareness and knowledge regarding the safety and advantages of this drug among both gynaecologists and new mothers, the fear of side effects, the fear of effects on breastfeeding, and difficulty in procuring the pills. In each PDSA cycle, there was an increasing trend in the percentage of women choosing centchroman (Figure 2). Following the first intervention, when charts providing information on Chhaya were displayed at the postnatal clinic, the acceptance rates improved from 2.9% to 15.3%. After the second intervention, which involved educating and creating awareness about centchroman by the nursing staff working on the study, the acceptance rates improved drastically to 56.3%. It further increased to 78.2% after the third PDSA cycle, which involved resident doctors and faculty in postpartum contraceptive counselling sessions.

During the next 12 weeks of follow-up, the overall rate of acceptance for centchroman was 81.7%. It was possible to sustain this high rate of acceptance of centchroman among the public due to the continued effort of the staff and resident doctors in the postpartum clinic. The telephonic follow-up of the women accepting Chhaya as a postpartum contraceptive was done by the resident doctors posted in the family planning clinics to assess the rate of continuation. All women responded to telephonic conversations when contacted periodically for follow-up. It was found that the rates of continuation at one month, three months, and six months after acceptance were 96.7%, 89.5%, and 78.6%, respectively. The dosing schedule of centchroman-twice weekly for the first three months, followed by weekly-made it a convenient choice for most women. Among those who discontinued, the major reasons was the preference for a copper intrauterine device or medroxyprogesterone acetate injections as methods of long-acting reversible methods of contraception.

## Discussion

Almost 33% of married women of reproductive age in India do not use any methods of contraception [1]. Postpartum contraception is an effective method for ensuring adequate contraception coverage and reducing unmet needs. Centchroman, also known as ormeloxifene, is a selective oestrogen receptor modulator (SERM), which is a type of medication that works on the oestrogen receptors. It is a non-steroidal oral contraceptive that is taken twice weekly for the first three months, then once weekly. Centchroman was introduced into the family planning basket of contraceptives but is underutilised as a postpartum spacing method despite its safety and convenient dosing schedule. Recent studies have demonstrated that centchroman has higher satisfaction and continuation rates than postpartum intrauterine devices and progesterone-only pills [1,4]. No untoward side effects have been reported with centchroman use till now [2]. The most common side effects are delayed and irregular menstrual cycles, as well as a few episodes of unwanted and unpredictable spotting [5]. A high acceptance rate for centchroman has been demonstrated in some studies as a spacing method [6]. It is a safe alternative where steroidal agents are contraindicated as there is no effect on blood pressure, the coagulation pathway, or lipid or carbohydrate metabolism. It has been demonstrated that the majority of cycles reverted to regular within six to nine months of its use, and the return of fertility upon discontinuing the agent was prompt [6]. Besides contraception, centchroman has also proven to be of benefit in the management of various other conditions like dysfunctional uterine bleeding, mastalgia, fibroadenoma, and the prevention of breast cancer [7]. Because of its low oestrogenic activity, it also has anti-osteoporotic and cardioprotective properties [3]. 

Despite being aware of the availability of spacing methods and having a desire for spacing, a large proportion of women do not utilise any methods of spacing due to fears and misconceptions [7]. There is an urgent need to optimise counselling in the postpartum period and provide effective contraception. The underutilization of Chhaya as a postpartum contraceptive is most likely due to a lack of knowledge about the drug among women and health care workers, as well as apprehension about its use. This QI project successfully increased its acceptance rate among postpartum women through the implementation of three PDSA cycles. Post-intervention follow-up showed continued improvement in the overall acceptance rate. Even though on long-term follow-up at six months, there was a slight drop in the acceptance rates to 78.6%, there was a satisfactory improvement in the acceptance compared to the pre-intervention period. Counselling by doctors seemed to be more effective, probably because of the trust factor and also because the doubts and queries regarding its safety profile and use were more efficiently handled. We plan to conduct regular audits every six months to monitor and ensure the continuation of holding the gains arising out of this QI initiative. 

The strength of our study is that it was the first QI project aimed at improving the acceptance rates of centchroman as a postpartum contraceptive. It also helped improve the knowledge of the family planning team members, which is vital to future counselling strategies. Future QI initiatives can help improve contraception coverage and reduce unintended pregnancies and their complications.

Limitations 

A major limitation of this study is that it is a single-centre study. Multicenter projects conducted in various parts of the country can bring to light other aspects related to postpartum contraception and strategies to improve the use of spacing methods. The other major limitation was that partner counselling was not possible due to constraints in time and manpower in a busy outpatient department. 

## Conclusions

Improvement in centchroman acceptance rates needs effective counselling to alleviate the fear that couples have regarding this relatively new drug. It was through the collaborative effort of proper counselling and meticulous follow-up by the doctors, nursing staff, and counsellors that a good acceptance rate could be sustained over time. Future QI initiatives can focus on other aspects, such as strategies for the easier provision of centchroman or reducing discontinuation rates to ensure sustained adherence. It is time to take the cafeteria approach and offer a basket of choices to women for postpartum contraception. Centchroman, though new, can be a good alternative to progestin-only pills owing to its less stringent dosing schedules and lack of hormone-related side effects.
